# External quality assessment of malaria microscopy in the Democratic Republic of the Congo

**DOI:** 10.1186/1475-2875-10-308

**Published:** 2011-10-18

**Authors:** Pierre Mukadi, Philippe Gillet, Albert Lukuka, Ben Atua, Simelo Kahodi, Jean Lokombe, Jean-Jacques Muyembe, Jan Jacobs

**Affiliations:** 1 Institut National de Recherche Biomédicale (INRB), Kinshasa, Democratic Republic of the Congo; 2 Department of Clinical Sciences, Institute of Tropical Medicine (ITM), Nationalestraat 155 B 2000 Antwerp, Belgium; 3 Programme National de Lutte contre le Paludisme (PNLP), Kinshasa, Democratic Republic of the Congo; 4 Programme National de Lutte contre la Tuberculose (PNLT), Kinshasa, Democratic Republic of the Congo; 5 Faculté de Médecine, Université de Kinshasa, Kinshasa, Democratic Republic of the Congo; 6 Department of Medical Microbiology, School for Public Health and Primary Care: CAPHRI, Maastricht University Medical Center, The Netherlands

## Abstract

**Background:**

External quality assessments (EQA) are an alternative to cross-checking of blood slides in the quality control of malaria microscopy. This study reports the findings of an EQA of malaria microscopy in the Democratic Republic of the Congo (DRC).

**Methods:**

After validation, an EQA slide panel and a questionnaire were delivered to diagnostic laboratories in four provinces of DRC. The panel included three samples for diagnosis (sample 1: *Plasmodium falciparum*, 177,000/μl, sample 2: *P. falciparum*, 2,500/μl, sample 3: no parasites seen), one didactic sample (Howell-Jolly bodies) and one sample for assessing the quality of staining. Participating laboratories were addressed and selected through the network of the National Tuberculosis Control Programme. Participants were asked to return the responses together with a stained thin and thick blood film for evaluation of Giemsa stain quality.

**Results:**

Among 174 participants (response rate 95.1%), 26.2% scored samples 1, 2 and 3 correctly and 34.3%, 21.5% and 5.8% of participants reported major errors in one, two or three samples respectively. Major errors included reporting "no malaria" or "non-*falciparum *malaria" for *Plasmodium falciparum*-positive samples 1 and 2 (16.1% and 34.9% of participants respectively) and "*P. falciparum*" for *Plasmodium *negative sample 3 (24.0%). Howell-Jolly bodies (didactic sample) were not recognized by any of the participants but reported as "*P. falciparum*" by 16.7% of participants. With parasite density expressed according to the "plus system", 16.1% and 21.5% of participants scored one "+" different from the reference score for samples 1 and 2 respectively and 9.7% and 2.9% participants scored more than two "+" different. When expressed as counts of asexual parasites/μl, more than two-thirds of results were outside the mean ± 2SD reference values. The quality of the Giemsa stain was poor, with less than 20% slides complying with all criteria assessed. Only one quarter of participants purchase Giemsa stain from suppliers of documented reliability and half of participants use a buffered staining solution. One third of participants had participated in a formal training about malaria diagnosis, half of them earlier than 2007.

**Conclusion:**

The present EQA revealed a poor quality of malaria microscopy in DRC.

## Background

The detection of *Plasmodium *parasites by light microscopy is still the primary method of malaria diagnosis in most health care facilities throughout the world [1]. Quality control programmes are a prerequisite of competent microscopy. The World Health Organization (WHO) recommends the cross-checking of blood slides: a sample of routine blood slides is sent to the reference laboratory, where it is checked for accuracy. External quality assessment (EQA) programmes (also referred to as "proficiency testing") are an alternative approach: in such programmes, the reference laboratory sends stained blood film samples to the peripheral laboratories, which assess them and submit a report, after which they are given feedback about the correct results and their own performance [2].

In the Democratic Republic of the Congo (DRC), malaria is endemic and 97% of the population is living in areas of stable malaria transmission. Parasite-based diagnosis of malaria is mainly performed by microscopy. Due to difficult economical and logistic conditions, countrywide programmes of quality control of malaria microscopy do not exist in DRC and till now, EQA programmes on malaria microscopy have been only sporadically organized and were limited to the capital (Kinshasa) [3]. In 2010, the National Malaria Control Programme (Programme National de la Lutte contre le Paludisme, PLNP) organized an EQA session on malaria microscopy through its national reference laboratory (Institut National de Recherche Biomédicale, INRB) and the network of laboratories participating in the national programme of tuberculosis control (Programme National de la Lutte contre la Tuberculose, PNLT). The present study reports the results of this EQA session.

## Methods

### Participants

The EQA session was organized in 2010 and addressed clinical laboratories subscribing to the EQA sessions on parasitology of INRB as well as those involved in the network of PNLT as deployed in four provinces: Kinshasa, Bas- Congo, Katanga and the Oriental Province. In addition, the malaria reference laboratories at the provincial level (n = 11) were included. Laboratories were categorized as hospital-based (n = 72), referral health centre (n = 36), health centre (n = 44) and others (n = 11). Table 1 gives an overview of the participants who were located in areas of hyper- of holo-endemic malaria transmission, with *Plasmodium falciparum *accounting for more than 95% of malaria infections.

**Table 1:** Diagnostic laboratories participating in the EQA session on malaria microscopy

	Provinces					Estimated % nationwide coverage
Participants	Bas-Congo	Kinshasa	Province Orientale	Katanga	Total	
Provincial Reference Laboratories	1	1	1	1	11*	100,0
Hospitals	24	32	4	12	72	9.9
Referral Health Centres	22	11	0	3	36	1.0**
Health Centres	6	1	32	5	44	
Private Laboratories	0	10	0	1	11	N.D.
Total	53	55	37	22	174	1.9

* Including the Provincial Reference Laboratories of the other seven provinces in DRC

** Combined on a total of 8,266 centres

N.D. = no data^2^

### Samples

The EQA panel consisted of five samples. Three samples were prepared as Giemsa-stained thick and thin blood film preparations with accompanying patient's history and white blood cell count/μl (Table 2). Participants were asked to report the diagnosis (malaria yes or no), *Plasmodium *species identification and parasite density. For parasite density, participants could express the value as asexual parasites/μl or as a "plus system" scale (range from "+" to "++++") [4,5], as both systems were endorsed by the national malaria control programme at the time of EQA session. A fourth sample was a thin blood film with red blood cells containing Howell-Jolly bodies, it was considered as a didactic sample. A fifth sample consisted of an unstained thin blood film: participants were asked to stain this blood film according to their usual procedure and return the sample. Finally, participants were asked to select a routinely processed thick blood film slide of their own laboratory and to return it together with the fifth sample. Both samples were used to assess the quality of the staining and thick blood film preparation as performed by the participants.

**Table 2:** Clinical information, *Plasmodium *species and parasite density of the samples submitted as part of the EQA session

Sample	Sample information	Reference result/Comment
1. Thick + thin blood film, Giemsa stained	Girl 9 years old, fever, pale, weakness, Leukocyte count 10,400/μl	*Plasmodium falciparum*, parasite density 177,000/μl or "++++"
2. Thick + thin blood film, Giemsa stained	Woman 54 years old, headache and fever, Leukocyte count 2,500/μl	*Plasmodium falciparum*, parasite density 86/μl or "+"
3. Thick + thin blood film, Giemsa stained	Man 57 years old, check-up visit	No parasites seen
4. Thin blood film, Giemsa stained	Woman, 39 years old, no clinical information	No parasites seen, presence of Howell-Jolly bodies
5. Thin blood film, unstained	Man, 36 years old, no clinical information	Sample to be stained by the participants and returned for scoring of staining quality
6. No sample	Stained thick blood film sample to be selected from routine samples by the participant	Sample to be sent to the reference lab for scoring of staining quality

The samples were prepared from left-overs of routinely drawn EDTA-blood samples obtained from patients presenting at INRB and prepared and processed according to the recommendations of PNLP and the WHO malaria microscopy quality assurance manual [1,6]. Briefly, thin and thick blood films of each sample were applied on a single slide (pre-cleaned slides, Menzel-Gläzer Braunschweig, Germany). Fixation of thin blood films was done with methanol (Panreac, Barcelona- Spain), and thin and thick blood films were stained with Giemsa pH 7.2 (MERCK, Darmstadt- Germany) and examined by light microscopy using a × 1,000 magnification. Parasite densities were estimated by counting asexual parasites against 200 white blood cells in the thick blood film and converting this number to parasites/μl using the actual white blood cell count as calculated microscopically in a Neubauer chamber [7]. To compensate for technical and observational variations in the parasite count, three expert microscopists counted the parasite densities on 10 slides for each *Plasmodium*-positive sample, and mean ± SD counts were calculated. In addition, parasite densities were assessed according to the "plus system", *i.e*. a semi-quantitative scale estimating the numbers of asexual parasites per high-power fields [5]. Species identification was confirmed by *Plasmodium*-specific polymerase chain reaction (PCR) [8,9]. Stained samples were allowed to dry, packed in plastic boxes and stored for a maximum of 90 days before shipment.

### Questionnaire

In addition to the samples, a questionnaire was submitted. The questionnaire addressed the following issues: numbers of requests for malaria diagnosis, slide positive ratio, staining procedures and training. In addition, participants were surveyed about the use of malaria rapid diagnostic tests (RDTs).

### Shipments

Samples and questionnaire were packed in plastic slide boxes (Slide mailer, Menzel-Gläser, Braunschweig, Germany) and shipped in protected envelopes (Air Pro 4, Propac, Malmo, Sweden) according to UN 3373 recommendations (i). For Kinshasa province, envelopes were transported and on-site delivered by car by an INRB collaborator. Samples for the provinces were shipped by private air carrier to the provincial airports (Kisangani, Lubumbashi and Boma/Kimpese in Oriental Province, Katanga and Bas-Congo respectively) where they were received by the provincial coordinators of PNLT or their representatives. Next, they were transported by car and hand-delivered to the participating laboratories. The results and questionnaire forms, as well as the slides of samples 5 and 6 were collected again by the PNLT representative and shipped to INRB.

### Validation of samples and questionnaire

For validation of the samples, shipment and questionnaire, a pilot EQA session was organized among 20 laboratories of known reputation (ten in Kinshasa, four in Bas-Congo, and three in Katanga and Oriental Province each). These 20 participants were actively surveyed about the quality of the samples, instructions and survey. Their results were included in the analysis.

### Data entry and analysis

The results were entered in an Excel database (Microsoft Corporation, Redmond, Washington, USA).

In diagnostic practice in DRC, parasite densities are generally expressed according to the "plus system": therefore, answers in terms of this score were primarily considered. In addition, values expressed as asexual parasites/μl were categorized in comparaison to the mean reference count ± SD [10]. For evaluation of the quality of staining and sample preparations (Samples 5 and 6), returned samples were assessed by two microscopists and scored according to criteria from previous studies and WHO recommendations (Table 3) [1,11,12]. Discordant results were assessed by a third observer and the consensus result was considered.

**Table 3:** Results for the quality of staining of thin and thick blood film samples stained by the participants

Sample	Criteria	Numbers (%)
Sample 5: Thin blood film supplied by EQA, stained by participant and returned to the reference laboratory (n = 163)	No Giemsa stain precipitates observed	107 (65.6%)
	Red blood cells stained grey-pink	147 (90.2%)
	Chromatin of lymphocytes stained purple	60 (36.8%)
	Granules of neutrophils stained pink	100 (61.4%)
	Complies with all criteria mentioned above	16 (9.8%)

Sample 6: Routinely stained thick blood film of the participant submitted to the reference laboratory (n = 155)	Correct dimensions (> 1 cm) and thickness of the film	110 (71.0%)
	Complete hemolysis of the red blood cells	118 (76.1%)
	No Giemsa stain precipitates observed	60 (38.7%)
	Good contrast between nucleus and cytoplasm	70 (45.1%)
	Complies with all criteria mentioned above	30 (19.4%)

Continuous variables were assessed for significance using the Student's t-test. Differences between proportions were tested for significance using the Pearson's Chi-square test or, in case of small sample sizes, a two-tailed Fisher's exact test. Trends in proportions were assessed using Chi-square test for trend.

The results for the microscopic diagnosis (samples 1, 2 and 3) were categorized as correct, or with minor and major errors. Major errors included (i) incorrect diagnosis of malaria, *i.e*. reporting "negative" in the case of a *Plasmodium*-positive sample and vice versa, (ii) not mentioning the presence of *P. falciparum *(either reporting non-*falciparum *species in the case of *Plasmodium falciparum *or no species identification at all) and (iii) parasite densities scored more than two "+" different from the reference result or not scored at all. A minor error was defined as a parasite density differing one "+" from the reference result.

## Results

### EQA sessions

The pilot EQA session was performed between August and September 2010, the regular EQA session was performed during the period September-November 2010. Results of 174 out of 183 laboratories were received, with a response rate of 95.1%. Median duration of shipment from INRB to participants was 9 days (range 1 - 67 days), and the reports were returned within a median delay of 4 days (range 1 - 25 days).

### Results for the microscopic analysis of the samples

Tables 4, 5 and 6 present the results for samples 1, 2 and 3 respectively. For sample 1 (*P. falciparum*, 177.000/μl) 59.2% of participants scored correct results and another 16.1% reported minor errors, all but one were errors in parasite density (reporting "+++" instead of the expected "++++" score, 17.8%). Nearly a quarter (n = 43, 24.7%) of participants reported major errors, these errors included (i) reporting "no malaria" (5.2% of participants), (ii) "non-*falciparum *malaria" (9.8%), and (iii) "+" or "++"or no parasite density at all (8.6%); two participants (1.1%) combined the latter two errors. Sample 2 (*P. falciparum*, 86/μl) was scored correct by less than half (41.9%) of participants: 37 (21.5%) participants reported minor errors, all but one scoring "++" for parasite density instead of the expected "+". Sixty-three (36.6%) of participants reported major errors including (i) "no malaria" (16.3%), (ii) "non-*falciparum *malaria" (18.6%), and parasite densities above "++" (2.9%). Two-thirds of participants scored sample 3 (no parasites observed) correct. Major errors among the remaining third included reporting of *P. falciparum *malaria (24.10%) with 9 (5.2%) of participants reporting parasite densities above "+". None of the participants mentioned the presence of Howell-Jolly bodies in sample 4. By contrast, for this parasite-negative sample, 24 (16.7%) of participants reported the presence of *Plasmodium *parasites, mostly *P. falciparum*.

**Table 4:** Results for sample 1: *P.falciparum*, parasite density 177.000/μl or "++++". Valid answers of 174 participants were included

		Parasite density ("plus system")					Total
			
Reported result	No parasites observed	Not reported	+	++	+++	++++	n (%)
*P. falciparum*		1	5	9	27**	103*	145 (83.3%)
*P. falciparum *and *P. malariae *						1**	1 (0.6%)
*P. malariae*		1			1	2	4 (2.3%)
No species identification reported				1	3	11	15 (8.6%)
No parasites observed	9						9 (5.2%)

Total	9	2	5	10	31	116	174 (100%)

*Correct result, including one result reported as "+++++"

**Minor error

**Table 5:** Results for sample 2: *P.falciparum*, parasite density 86/μl or "+". Valid answers of 172 participants were included

	Parasite density ("plus system")					Total
		
Reported result	No parasites observed	Not reported	+	++	+++	n (%)
*P. falciparum*		2	72*	36**	3	113 (65.7%)
*P. vivax *and *P. falciparum*			1**			1 (0.6%)
No species identification reported			27	5		32 (18.6%)
No parasites observed	26					26 (15.1%)

Total	28	2	100	41	3	172 (100.0%)

*Correct result

**Minor error

**Table 6:** Results for sample 3: No parasites observed

		Parasite density ("plus system")					Total
			
Reported result	No parasites observed	Not reported	+	++	+++	++++	n (%)
No parasites observed	116*						116 (66.6%)
*P. falciparum*		1	35		4	1	41 (23.6%)
No species identification reported		1	11	2	1	1	16 (9.2%)
*P. falciparum*, gametocytes		1					1 (0.6%)

Total	116	3	46	6	2	1	174 (100.0%)

Valid answers of 174 participants included

*Correct result

Among the participants who had valid answers for all three samples (n = 172), 26.2% scored all three correctly; 34.3%, 21.5% and 5.8% had major errors for one, two and all three samples respectively.

### Parasite density expressed as parasites/μl

**Figure 1:**
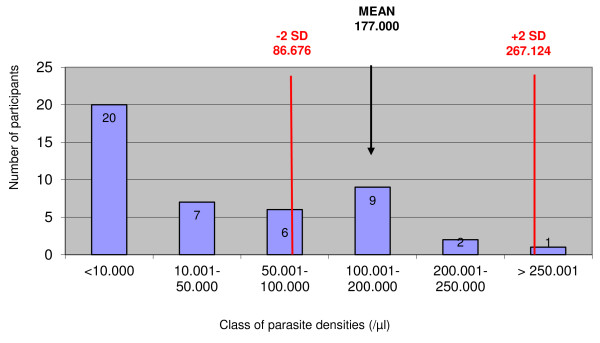
**Distribution of parasite densities expressed per μl of blood for sample 1 and 44 participants**. Mean and SD values refer to the expected result as counted by expert microscopy.

**Figure 2:**
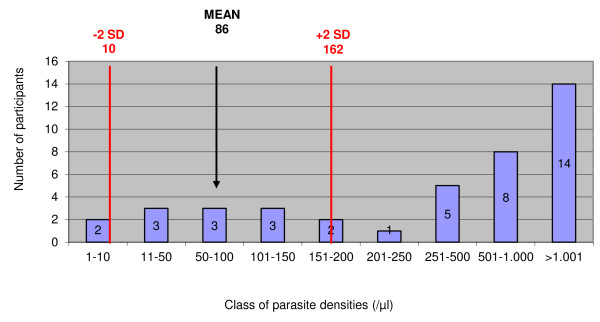
**Distribution of parasite densities expressed per μl of blood for sample 2 and 41 participants**. Mean and SD values refer to the expected result as counted by expert microscopy.

In addition to their report expressed as "plus system", 44 and 41 participants reported parasite densities as numbers of asexual parasites/μl for samples 1 and 2 respectively. Figures 1 and 2 display the distribution of the parasite densities with respect to the mean ± SD reference values. For sample 1, reported parasite densities ranged between 32 and 2,700,000/μl. Thirty-two (72.7%) participants reported a parasite density > 2 SD above or below the reference value, with 22 (49.9%) and 1 (2.3%) participants reporting a value 10 times higher or lower the mean reference value. For sample 2, these numbers were 32 (78.1%), 2 (4.9%), 16 (39.0%) respectively, and parasite densities ranged between 1 and 32.000/μl.

### Quality of Giemsa staining as scored on thin and thick blood film preparations

Table 3 lists the scores for the quality of the Giemsa staining for sample 5 (thin blood film supplied by EQA) and sample 6 (thick blood film from the participants' routine diagnosis). Although some scores for the individual criteria were high, the overall quality was modest, with only a minority (< 20%) of returned slides complying with all criteria assessed.

## Results of the questionnaire

**Figure 3:**
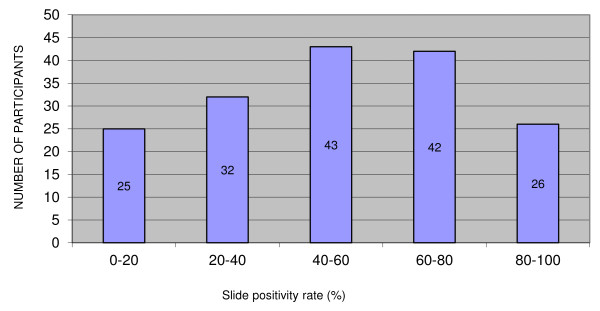
**Distribution of slide positive rates among the participants (168 valid answers, data represent numbers)**.

One third (32.7%) of participants had processed less than 100 samples during the month before the EQA and 58.3% had processed less than 200 samples. A total of 25 (14.9%) of participants reported a slide positivity ratio below 20%, whereas two-thirds (66.0%) declared slide positivity ratios ≥ 40%, among which 26 (15.5%) ≥ 80% (Figure 3).

Nearly two-thirds (63.8%) of participants had never participated in a formal training on malaria microscopy, and among those who did, more than half were trained earlier than 2007. The situation was worst for the health centres and reference health centres (no training for 69.8% and 75.0% of them repectively) than for the Provincial Reference Laboratories and referral hospitals (no training for 45.5% and 52.5% of them respectively) but this difference was not statistically significant (p = 0.136).

Participants were asked about their supply for Giemsa stain and the use of buffer solution. The vast majority (87.9%) purchased Giemsa stain as a stock solution. As to the choice of supplier, only one quarter (25.3%) of participants procured it from suppliers of documented reliability like central purchasing services, non-governmental organizations and the national malaria control programme, while one third (35.6%) relied on "fournisseurs ambulants", *i.e*. private vendors who visit and deliver on site. This reliance was more apparent among the reference health centres (22/36, 61.1%). For preparation of the working solution, half (52.3%) of participants used a buffer solution, 29.9% and 17.8% used distilled water and regular water (mostly tap water) respectively. One quarter (24.7%) of participants used RDTs, half of them since one year or less. The majority (35, 81.4%) used Paracheck (Orchid Biomedical Systems, Goa, India).

### Associations between performance and different parameters

**Figure 4:**
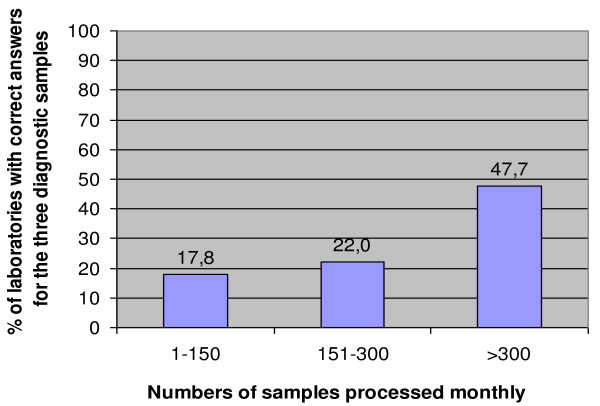
**Numbers of samples processed monthly by the participants associated with the percentage of correct answers for the three diagnostic samples**.

EQA performances in terms of correctness of answers for the three diagnostic samples correlated with the numbers of samples processed monthly (p < 0.001, Figure 4) but not with other factors such as province, training during the previous years or quality and origin of the Giemsa stain. In addition, there was a tendency towards better results related to hierarchy of structure, with 16.3% of health centres, 20.0% of reference health centres, 34.7% of hospitals and 45.5% of provincial laboratories replying correct results for all three samples (p = 0.064).

## Discussion

Among 174 diagnostic laboratories in DRC participating on an EQA session on malaria microscopy, 26.2% scored all three samples correctly and 34.3%, 21.5% and 5.8% reported major errors in one, two or all three samples respectively. Major errors included missing the diagnosis of *P. falciparum *malaria and diagnosis of malaria in negative samples as well as errors in estimation of parasite density. Most participants had serious problems for the purchase and the preparation of the Giemsa stain solution and two-thirds were not formally trained in malaria microscopy.

### Errors in diagnosis: false negative and false positive results

Major errors included reports of "no malaria" or "non-*falciparum *malaria" in the case of *P. falciparum*-positive samples (16.1% and 34.9% for samples 1 and 2 respectively): such false negative results may cause potentially lethal consequences and erode confidence of clinicians and community. Identification up to the level of *P. falciparum *has been noted as a difficulty in non-endemic settings too: a EQA session in Canada in 1995 recorded 27% errors in the species diagnosis of *P. falciparum*, and 21% failures were registered at an EQA session in the UK [12,13]. Further, 24.0% and 16.7% of participants in the present survey reported "*P. falciparum*" in the parasite negative samples 3 and 4 respectively: these false positive results lead to unnecessary treatment adding to costs side effects and distract the clinician from considering other causes of fever and disease. For comparison, EQA sessions in non-endemic sessions recorded false-positive results by 2% - 15% of participants and, in line with present observations, Howell-Jolly bodies, fibrin strands and stain granules were confused with blood parasites [13,14].

### Errors in estimation of parasite densities

With parasite density expressed according to the "plus system", about 20% and 10% of participants scored one "+" or ≥ "++" different from the reference value (considered as minor and major errors respectively). The quantification according to the "+" scale is subject to inconsistencies and WHO recommends it gradually replacement by counts of asexual parasites/μl. The latter system has only been introduced by PNLP since October 2010, which may explain in part for the poor results presently observed. However, the high numbers of apparent failures (> 10 × above or below the reference value) illustrates the need for clear procedures, training and follow-up during future EQA sessions [10]. Estimation of parasite densities has been revealed as problematic in several EQA sessions in non-endemic settings, with up to 39% failures [13,14].

### Reliable purchase of Giemsa stain and buffer

The quality of the Giemsa stain was poor, with < 20% of slides stained by the participants complying with all criteria assessed. Only one quarter of participants purchased Giemsa stain from traceable suppliers such as central purchasing services and the PNLP, while one third relied on private salespersons and only half of them used a buffered solution for the Giemsa stain. These observations may be explained by the difficult economic and logistic situation in DRC. Purchase policies and supplier evaluations are part of all quality assurance systems and essential when considering the Giemsa stain, for which quality-controlled production and pH monitoring are essential [4].

### Training and critical volume

Only one third of participants had attended a formal training about malaria microscopy, half of them earlier than 2007. Although slightly better at the central and intermediate levels, the situation was of concern too. Training is essential for maintaining competency and commitment of microscopists [4] and PNLP is actually developing trainings programmes. One third of participating laboratories processed less than 100 samples monthly. In addition, there was an association between correct scores for this EQA and the numbers of samples processed monthly. Although the numbers of laboratory staff involved in malaria diagnosis was not accounted, this provides indirect evidence towards the association between low exposure and poorer performance. For microscopists - provided correctly trained - the reading of at least 10 slides a month is recommended by WHO in order to maintain competence [4]. Further, the slide positivity rate varied widely: although till now this parameter was only used as in indirect indicator for malaria transmission and not for laboratory quality assessment [15,16]. The value of the slide positivity rate as an indicator of quality assurance may be explored. For instance, slide positivity rates ≥ 80% even in highly selected patients might be questioned for accuracy [17].

### Limitations and strengths of EQA sessions including the present one

The present EQA session undoubtedly suffered from limitations inherent to EQA methods: for instance, results for samples and questionnaires may reflect theoretical competence and knowledge rather than day-to-day performance [14]. A second limitation relates to the coverage of this EQA as less than 10% of hospitals and 1% of the health centres nationwide were included. Finally, the current participants probably represent the better laboratories, as participation was voluntary and directed to laboratories already involved in the EQA network of the PNLT. Logistic difficulties - a known limitation of EQA sessions in resource constrained settings [18] were behind this selection and approach, since transport infrastructures and internet communication in DRC are limited, and the actual number of participants was only reached by joining the network of PNLT with its regional collaborators.

As to their strengths, it should be noted that EQA sessions particularly when considered in an educational and non-punitive atmosphere offer a didactic stimulus and boost self-confidence and trigger towards implementation of a quality system, as participation is required for accrediting norms such as ISO 15189 for medical laboratories [2,19]. For the health authorities and control programmes in resource constrained settings, EQA sessions may be the first or unique information about nationwide laboratory performance, diagnostic practices and the quality of reagents or diagnostic tests [18,20-22]. The results of the present EQA provide benchmark information: this allows to monitor the progress in diagnostic performance through follow-up EQA sessions. In addition, EQA sessions are more cost-effective than cross-checking of slides [18]. Finally, the actual collaboration between the PNLP and PLNT is in line with the WHO recommendation of integrating malaria microscopy quality assessment with that of other microscopically diagnosed diseases such as tuberculosis [23,24].

### Place and role of malaria rapid diagnostic test, integration in quality assurance

The present results are in line with observations about inadequate quality of microscopy in field settings [25]. The input needed for corrective actions at all points of microscopy quality assurance will probably be very high; therefore one might tend to favor rolling out malaria rapid diagnostic tests (RDTs) at all levels of health care. Indeed, despite the fact that RDTs no give information about parasite density and species identification, their diagnostic accuracy in field settings may equal or exceed that of microscopy [26,27] and it is obvious that they are less demanding with regard training, equipment and infrastructure. PNLP is deploying RDTs in DRC since June 2010. As for malaria microscopy, malaria RDTs however need to be deployed in a quality assured environment at all levels [28-34].

## List of abbreviations

DRC: Democratic Republic of the Congo; EDTA: Ethylene Diamine Tetra-acetic Acid; EQA: external quality assessments; INRB: Institut National de Recherche Biomédicale; ITM: Institute of Tropical Medicine; *P: Plasmodium*; PCR: polymerase chain reaction; PNLP: Programme National de Lutte contre le Paludisme; PNLT: Programme National de Lutte contre la Tuberculose; RDT(s): Rapid Diagnostic Test(s); SD: standard deviation; UN: United Nations; WHO: World Health Organization.

## Competing interests

The authors declare that they have no competing interests.

## Authors' contributions

PM, PG, AL and BA designed the external quality assessment. PM and AL carried out the try-out EQA session. PM, SK and JL carried out the formal EQA session. PM, PG and JJ analyzed and interpreted the results and drafted the manuscript. All authors drafted and critically reviewed the manuscript and approved the final manuscript.
